# A combined approach exploring gene function based on Worm-Human Orthology

**DOI:** 10.1186/1471-2164-6-65

**Published:** 2005-05-06

**Authors:** Ivica Tamas, Emily Hodges, Patrick Dessi, Robert Johnsen, Ana Vaz Gomes

**Affiliations:** 1 Department of Molecular Biology and Functional Genomics, Stockholm University, Sweden; 2 Center for Genomics and Bioinformatics, Karolinska Institute, Stockholm, Sweden; 3 Södertörn University College, Stockholm, Sweden; 4 Department of Molecular Biology and Biochemistry, Simon Fraser University, Burnaby, Canada

## Abstract

**Background:**

Many aspects of the nematode *Caenorhabditis elegans *biology are conserved between invertebrates and vertebrates establishing this particular organism as an excellent genetic model. Because of its small size, large populations and self-fertilization of the hermaphrodite, functional predictions carried out by genetic modifications as well as RNAi screens, can be rapidly tested.

**Results:**

In order to explore the function of a set of *C. elegans *genes of unknown function, as well as their potential functional roles in the human genome, we performed a phylogenetic analysis to select the most probable worm orthologs. A total of 13 *C. elegans *genes were subjected to down- regulation via RNAi and characterization of expression profiles using GFP strains. Previously unknown distinct expression patterns were observed for four of the analyzed genes, as well as four visible RNAi phenotypes. In addition, subcellular protein over-expression profiles of the human orthologs for seven out of the thirteen genes using human cells were also analyzed.

**Conclusion:**

By combining a whole-organism approach using *C. elegans *with complementary experimental work done on human cell lines, this analysis extends currently available information on the selected set of genes.

## Background

Many aspects of the nematode *Caenorhabditis elegans *biology are conserved between invertebrates and vertebrates establishing this particular organism as an excellent genetic model. Because of its small size, large populations and self-fertilization of the hermaphrodite, functional predictions carried out by genetic modifications as well as RNAi screens, can be rapidly tested. This is an obvious advantage when compared to the increasingly complex *Drosophila *and mouse genomes. Therefore, the nematode *C. elegans *has emerged as an excellent entry point to begin to address these predictions. There are currently two main approaches in *C. elegans *to investigate gene function on a genomic scale using reverse-genetics. The first one is based on RNA-mediated interference (RNAi) where "functional knock-outs" of a particular gene can be studied and phenotypes identified. The other is a PCR -based technology to identify genetic mutants in a mutagenized library. Complementary expression data can also be acquired from microarray data or by Serial Analysis of Gene Expression (SAGE) to identify genes that are co-expressed or up/down regulated under defined conditions. These methods can be directly combined, on a particular set of genes, to provide a comprehensive description of the gene's expression patterns and functions [1]. In addition, results can be refined using independent screens of other experimental systems, for example human cell lines.

RNAi based on the introduction of double-stranded RNA (dsRNA) is the method that results in specific inactivation of the corresponding gene through the degradation of endogenous mRNA. It was originally described in *C. elegans *[2,3] and has become the main reverse-genetics tool for determining the function of specific genes. Several large-scale studies involving *C. elegans *[4-7], subjected approximately 90% of the 19,427 predicted genes to down regulation via RNAi. Moreover, individual clones of the entire RNAi feeding library described in Fraser et al. [5] can be ordered directly. In addition, in vitro synthesis of ~21-nt small interfering RNAs to mediate gene-specific suppression in mammalian cells have been developed in order to extend this particular technique to higher eukaryotes [8].

80% of *C. elegans *genes have human homologs [9]. As long as we are able to establish orthology, information obtained for a gene sequence in one organism is potentially transferable to the other [10]. Nevertheless, since the human and the worm genomes are phylogenetically very distant, in many cases these sequences are only able to produce poorly supported trees. Moreover, position of individual branches may not be conserved between different algorithms for finding distance trees. Phylogenetic reconstructions involving human/worm sequences in a number of cases are unable to resolve their exact phylogenetic past. In the case of orthology assignments they provide only an indication for its existence.

Moreover, sequence orthology does not necessarily imply the same function. However, genes shown to be descendants of the same gene (orthologous genes) have, in general retained the same function over the course of evolution [10]. If so, it is important to select for analysis only the genes where a clear orthology assignment can be established.

Based on a collection of uncharacterized protein families derived from a comparison of three available genomes including *H. sapiens*, *C. elegans *and *D. melanogaster*, an integrated in-house program for functional gene annotation was initiated. As part of this annotation effort, a set of genes originating from this collection of novel protein families was selected for functional analysis in both *C. elegans *and human cell lines.

In this study novel protein families are explored using a diverse set of technologies involving both *in silico *and experimental analysis with the intention of identifying interesting gene candidates showing evidence of highly conserved function which may serve as potential drug targets in the future.

We have sought to identify, through phylogenetic analysis, the most likely worm orthologs for a set of human genes. Expression patterns and RNAi data in the worm were obtained, as well as corresponding data using human cell lines.

## Results and Discussion

### Phylogenetic analysis: the most likely ortholog?

Multiple gene duplication followed by massive gene loss and acquisition of novel functions has been shaping the evolution of distant organisms. In a search for the most likely orthologs, special attention must be paid to the fate of duplicated genes when related to speciation. Duplicated genes tend to evolve in different patterns following the duplication event arising from different functional constrains [11].

According to Remm et al. and Sonnhammer and Koonin, out-paralogs are paralogs that predate speciation [12,13]. By contrast, in-paralogs are genes that arose after speciation. Therefore, all in-paralogs are considered potential functional orthologs. Using translated sequence we compare maximum parsimony trees to neighbor joining counterparts. Genes for which the position of the branches (in particular the branches leading to *C. elegans *and its closest human homolog) was conserved between both algorithms chosen for finding distance trees (Neighbor joining versus Maximum Parsimony), were selected for the experimental study (Tab. 1.). This criterion has been used in order to eliminate genes producing unstable topologies caused by duplications or a generally weak phylogenetic signal. Also, in this way it was possible to infer the homolog which most likely retained the ancestral gene function.

**Table 1:** Wormbase ID numbers and accession numbers for the analyzed set of *C. elegans *genes and their identified human homologs.

***C. elegans***		**Human ortholg**	
**Worm base ID**	**Description**	**Acession no.**	**Description**

T24D1.1	Chondroitin synthase	NP_055733	Chondroitin synthase 1
F23C8.6	Predicted coding sequence	NP_065145	CHMP1.5 protein
F38H4.7	Predicted coding sequence	AAK25825	BTB/POZ domain containing protein 1
C05C8.6	Predicted coding sequence	NP_060267	BTBD2 protein
C01A2.4	Predicted coding sequence	NP_054762	Hypothetical protein
C11D2.4	Predicted coding sequence	NP_115683	C9orf64 protein
F41D9.1	Predicted coding sequence	NP_056520	Hypothetical protein
C47D12.2	Predicted coding sequence	NP_060123	Dyggve-Melchior-Clausen syndrome protein
ZK795.3	Predicted coding sequence	NP_219484	U3 snoRNP protein 4 homolog
C17E4.3	Predicted coding sequence	NP_848545	Hypothetical protein MGC48332
B0379.4a	Predicted coding sequence	AAP34400	HYA22 protein. May be tumor supressor
C09D4.1	Predicted coding sequence	NP_060261	Feline leukemia virus subgroup C receptor-related protein 2
Y45F10A.6a	Predicted coding sequence	BAA74905	Hypothetical protein KIAA0882

**Figure 1:**
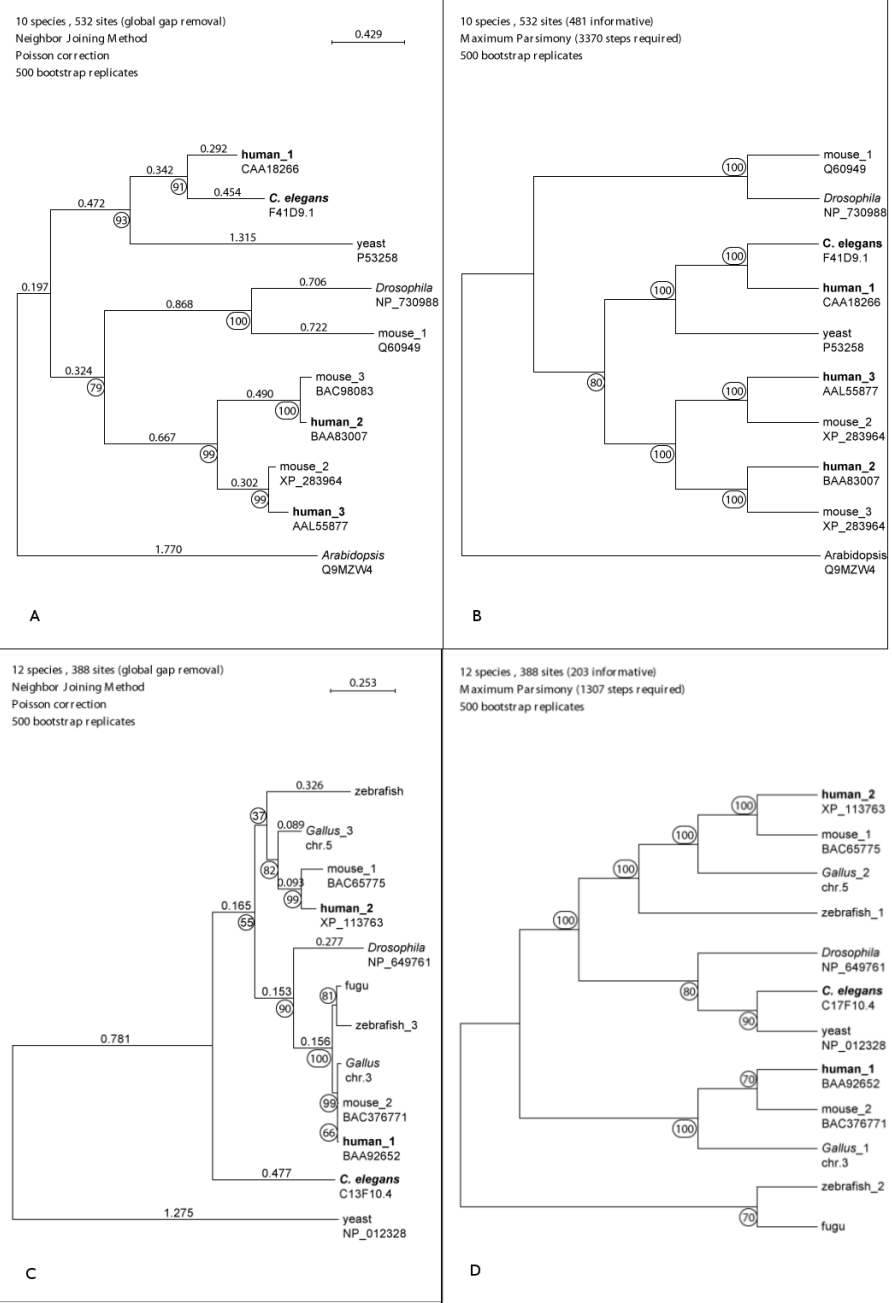
**Maximum parsimony and Neighbor joining trees for F41D9.1 (A, B) and C13F10.4 genes (C,D)**. Illustration of the criteria that has been applied in order to select the genes. Only genes able to produce trees as shown in A and B (F41D9.1) were subjected to experimental work.

Unique orthologous gene pairs are difficult to identify using standard similarity searches, as multiple candidate genes are typically obtained. Our trees typically contain two or more human homologs showing significant sequence similarity to a single worm gene (Fig. 1). Therefore, we selected candidate genes by choosing genes that produced trees that allow for identification of single human sequences as the most probable orthologs. These trees clearly discriminate out-paralogs or other in-paralogs (Fig. 1A and 1B). In effect, well-supported trees showing a one-to-one relationship between human and worm sequence were selected. However, since the purpose of this analysis was to identify genes that most likely retained the same function rather than orthology *sensu stricto*, it is possible that in some cases a paralog (in-paralog) was selected as the most promising ortholog. In other words, the *C. elegans *genes that we identified are either true orthologs or the best in-paralogs to the corresponding human genes [12]. Despite the fact that the exact type of orthology encountered in individual trees could not always be identified, we find this level of resolution satisfactory for the purposes of this study.

Thus, phylogenetic analysis (Fig. 1A and 1B) predicts F41D9.1 (Q94222) to have a similar function to human CAA18266. The tree also assigns other human sequences, namely BAA83007 and AAL55877, which are apparently a product of a duplication event in the mouse/human lineages, as either out-paralogs or in-paralogs. Regardless of their exact orthology/paralogy relation to the worm sequence, neither of them is the most probable ortholog.

To the contrary, a frequently encountered situation was obtained in the case of the worm gene C13F10.4 (Fig. 1C and 1D). The two human sequences though rather similar having a protein matrix distance of 0.61482 (Philip package), were easily separated in both the Neighbor Joining tree (NJ tree) and the Maximum Parsimony tree (MP tree). The NJ tree in particular demonstrates that the gene was duplicated in the animal series starting from *Gallus sp*. and upwards. Thus, in an ideal case, phylogenetic analyzes would correctly identify the exact copy of the duplicated gene in the human genome and assign it to a single *C. elegans *sequence as its true ortholog, as shown for F41D9.1 (Fig. 1A and 1B). The position of the other copy would point to its paralogous/in-paralogous relation to the worm sequence. However, the worm gene is either weakly associated with the branch leading to both human sequences (NJ tree, Fig. 1C) and it forms a separate branch with the XP_113763 human sequence (MP tree, Fig. 1D).

*C. elegans *genes and their most probable human orthologs are shown in the Tab. 1. Interestingly, due to the strict criteria we applied in order to select the most likely human orthologs, even C27A7.1, a known disease gene , which is regarded as orthologous to the human gene NPPASE (ENPP1; OMIM:173335) was not identified.

### Expression profiles and RNAi phenotypes

#### A distinct GFP expression: F41D9.1, C17E4.3, ZK795.3 and C09D4.1

**Figure 2:**
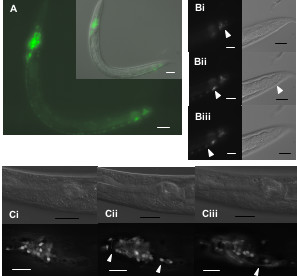
**F41D9.1::GFP expression. **Widespread through the neural system. Panel A shows the general expression pattern in an L1 stage animal. Many cells in the nerve ring, the ventral nerve cord (vnc) and the tail region express GFP. Panel B presents the tail region in greater detail, scanning through the animal at three focal planes from right (Bi) to left (Biii), with the ventral side facing down. A cluster of laterally symmetrical cells is visible in panels Bi and Biii, whereas in Bii cells of the vnc are visible. Magnification is 100×. Fig C presents 3 focal planes from dorsal (Panel Ci) to ventral (Panel Ciii) through the worm head, with the posterior pharyngeal bulb to the left. The GFP images have undergone deconvolution to increase resolution. Cells of the dorsal ganglion are visible in Ci, the retrovesicular ganglion is marked in Cii, and processes leading to it are indicated in Ciii. Scale bar represents10 μm.

Here we report GFP expression under the control of a putative promoter for F41D9.1 that occurs predominantly in many neural cells in the head around the posterior pharyngeal bulb, along the ventral nerve cord and in the tail (Fig 2). Head neurons include clusters in the dorsal and retrovesicular ganglia as well as the sensory amphid neurons. Expression is present in both larval and adult stages. No other phenotype was observed following down-regulation which is in accordance to previously published results by [14].

**Figure 3:**
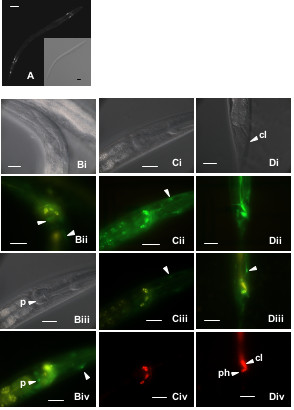
**C17E4.3::GFP reporter expression. **Present in the head and tail regions of larval stages. Panel Ai shows an overview of an L1 animal, with distinct cells near the anterior pharyngeal bulb as well as in the tail. Close up of the head reveals GFP expressing cells (Panel Bii), including muscle cells located in the posterior pharyngeal bulb (Panel Biv, labeled 'p'). Sheath/socket cells located at the anterior pharyngeal bulb are indicated in panels Biv, Dii and Diii. Dye-filling tests (see Materials and Methods) to stain sensory amphid (head) and phasmid (tail) neurons are shown in panels C and D. The amphids are specifically visualized in panel Civ (red fluorescence), and under an FITC filter in Ciii (yellow fluorescence, not in nucleus) and green with an EGFP filter, and are not the same as the cells expressing the C17E4.3::GFP reporter (arrow in panels Cii and Ciii). In the tail, phasmids (labeled 'ph') are clearly seen in panel Div just below the anus (cl), but are not expressing GFP, which is instead present in several hypodermal cells (arrow panel Diii). Scale bar represent 10 μm.

The C17E4.3::GFP reporter is expressed in the developing embryo and L1 larval stages, in three distinct sheath/socket cells in the head region close to the anterior bulb of the pharynx and several cells around the anus (Fig 3). Expression is also seen in several pharyngeal muscle cells. Dye filling tests confirmed that the processes extending to the nose were not from amphid neurons, but rather from socket cells (Fig 3, panel C). The expression occurs exclusively in the developing embryo and L1 larval stage suggesting involvement of the gene in development. No RNAi phenotype was associated with this gene. The wild type phenotype, following down-regulation, was also obtained by Simmer et al. [7]. However, the results obtained by Piano et al. [15] show that silencing of the gene results in embryonic lethality. Nevertheless, inconsistent phenotypes have been obtained by different groups for several other genes [16].

**Figure 4:**
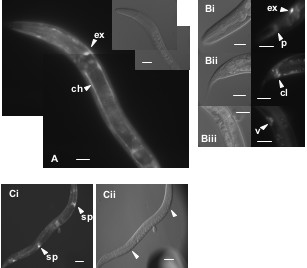
**ZK795.3::GFP reporter expression**. Evident in the excretory cell system (panel A) in about 20% of animals, but was always present in the excretory cell (labeled 'ex', panels A and Bi). The anal sphincter and/or depressor cell around the anus ('cl') also expressed GFP (panel Bii), as did the juvenile vulva ('v', panel Biii) and the spermathecae (panel C). Scale bar represents 10 μm.

ZK795.3::GFP expression pattern includes spermatheca, hypodermal cells, pharynx and the excretory cell and channels (Fig 4). In the L3 stage, expression was seen in the vulva, and in P6.p descendants.

**Figure 5:**
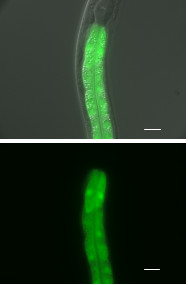
**C09D4.1::GFP expression. **Limited to the nuclei of intestinal cells. The top figure is an overlay of the DIC and GFP images. Scale bar represents 10 μm.

C09D4.1 had a simple expression pattern limited to intestinal cells in all developmental stages (Fig 5).

#### Visible RNAi phenotypes: T24D1.1, F23C8.6, ZK795.3 and B0379.4a

To investigate in detail the RNAi effects on growth, brood size and life span, we have closely followed a population of embryos (N2 and *rrf-3 *(NL2099) to full development at 20°C, in RNAi plates for corresponding genes with visible RNAi phenotypes.

*C. elegans *SQV-5 14162 protein (WP:CE 14162) encoded by T24D.1 gene is a chondroitin synthase that initiates/elongates chondroitin chains. This protein is also required for cytokinesis, gonad migration and vulval morphogenesis where it possibly promotes filling an extracellular space with fluid [17].

Following exposure to the corresponding dsRNA, a smaller F1 population size was observed when compared to the control animals in the both N2 and NL2099. F2 animals show a robust RNAi-induced phenotype including: sickly appearance, partial sterility, and small brood size. Lethality was observed in F3 embryos in the *rrf-3 *background at 20°C. The partial sterility among the F2 animals is in accordance with the reported  mutant phenotype (self-sterile hermaphrodites). All the phenotypes indicated above were reduced in the N2 background and also when grown at 15°C. Additional mutant phenotypes included squashed vulva (Sqv), and reduced L4 vulva invagination . However, according to Fraser et al. [5], down-regulation via RNAi did not produce a distinct phenotype. This is possibly due to the large-scale nature of their study and its focus on early developmental stages.

For F23C8.6, our results revealed slow growth (Gro) and uncoordinated behavior (Unc) which are both in agreement with the results obtained by Fraser et al. [5]. Our observations also confirmed larval arrest as reported previously [7]. As with T24D.1, ZK795.3 and B0379.4a, down-regulation of F23C8.6 did not affect life span. Gene function related to growth, larval development and locomotory behavior have been also inferred from the mutant phenotype .

Down-regulation of ZK795.3 affects the growth rate significantly in both wild type and the hypersensitive NL2099 strain (*rrf-3*). At 20°C growth was 10–15 times slower then the wild type control, slightly more pronounced in the *rrf-3 *strain. A large proportion of sterile F2 (Stp) animals was also observed. The minority of animals that were fertile produced very few eggs but these eggs were as viable as wild type. According to the previous studies down-regulation of the same gene has produced the following phenotypes: Gro, Emb, Stp, Lva [7,14,15].

Our results for down-regulation of B0379.4a are consistent with the data obtained by Kamath et al. [14]. RNAi resulted in an Egl phenotype that was more pronounced in the *rrf-3 *background. The involvement of the gene in oviposition was inferred from the mutant phenotype . The gene has orthologous sequences in both the human and mouse genomes that code for a small CTD phosphatase and nuclear LIM interactor-interacting factor 2, respectively.

RNAi on C05C8.6 has been reported by Simmer et al. as Emb, Lva and Lvl [7]. However, our results are consistent with another study published by Kamath et al. where no specific phenotype was observed [14]. The mutant phenotype of C05C8.6 points to its involvement in embryonic and larval development with a molecular function associated with protein binding .

Although a greater proportion of genes show specific RNAi phenotypes when using *rrf-3 *strain [7], our results point to an increase in the severity of the phenotypes following down-regulation in the *rrf-3 *background, rather than additional phenotypes.

### Subcellular localization in human cell lines

For seven of the human orthologs presented in this study, we cloned full-length open reading frames (ORFs), representing encoded cDNAs, into vectors containing V5 epitope sequences in order to generate recombinant fusion proteins for immunofluorescence detection. Because of the novelty of these proteins and the lack of available antibodies against such proteins, it was necessary to overexpress in human cell lines and subsequently detect with antibodies against the V5 fusion tag. Subcellular localizations were obtained in two human cell lines: HeLa and Human Embryonic Kidney 293 (HEK293). Endogenous expression for the proteins studied in the two cell lines was confirmed by RT-PCR.

**Figure 6:**
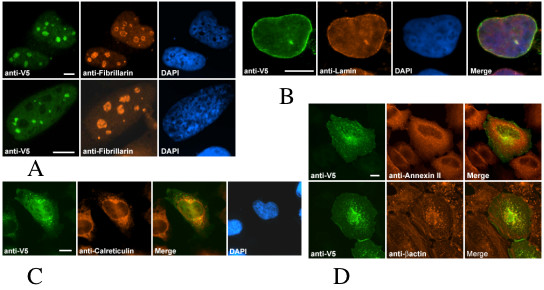
**Subcellular localization of recombinant fusion proteins in human cell lines. **Human protein NP_219484 (*C. elegans *protein ZK795.3) was detected in transfected HeLa cells with goat anti-V5 conjugated with FITC and co-localized with human anti-fibrillarin subsequently detected by donkey anti-rabbit Cy3 (A). Nuclei were stained with DAPI. This protein displays both a nucleolar (top row) and nuclear speckle (bottom row) pattern (A). NP_848545 (C. *elegans *protein C17E4.3) was detected in HEK293 cells with mouse anti-V5 and goat anti-mouse Alexa 488 (B). Cells were costained with rabbit anti-Lamin and donkey anti-rabbit Cy3 illustrating a colocalization with the nuclear envelope (B). Partial ER and nuclear distributions were observed for NP_115683 by co-detection with rabbit anti-calreticulin in HeLa cells (C). AAP34400.1 was detected in HeLa cells at the plasma membrane by co-staining with Annexin II (left panel, D) and a partial colocalization with beta-actin was also detected (right panel, D). Scale bars represent 10 μm.

NP_219484, human ortholog for ZK795.3, displays three distinct patterns: diffuse nuclear, nucleolar and nuclear foci (Figure 6A). We were able to confirm the nucleolar pattern by colocalization with fibrillarin, a protein predominantly found in nucleoli and cajal bodies. We were unable to determine the exact sub-compartment to which the nuclear foci belong. However, the pattern is strikingly similar to that of paraspeckles. The proteins, described as U3 snoRNP protein 4 homologs, belong to the IMP4 family of proteins which are small ribonucleoproteins involved in pre-ribosomal RNA processing. These results are consistent with those previously obtained in yeast and recently described in human [18]. Based on the interaction of these proteins with snoRNA in 60-80S RNP complexes and their likely involvement in pre-rRNA processing, it is possible to speculate that the preferred location of the Imp4 protein is both transient and dynamic throughout the nucleus, which would explain the three patterns we observe.

NP_848545 also described as hypothetical protein MGC48332 and predicted ortholog of worm protein C17E4.3 displayed a nuclear and perinuclear pattern covering the entire circumference of the nucleus but did not appear to be inside the nucleus. We were able to confirm the presence of this protein at the nuclear membrane by colocalization with Lamin, denoting a possible role for this protein as part of the nuclear envelope (Figure 6B). C3HC4 which contains a RING-finger motif, has been classified as the third member (MARCH-III) of the recently defined membrane-associated RING-CH protein family [19]. The same authors reported that proteins belonging to this family, including MARCH-III, characteristically contain two predicted C-terminal transmembrane domains, indicating a possible association with membrane-bound organelles. Furthermore, Bartee et al. concluded that localization of human MARCH III by fusion protein overexpression revealed a punctate pattern partially overlapping with cytoplasmic vesicles, specifically early endosomes. However, we were unable to confirm this finding. It is important to note that proteins are, in many cases, dynamic in location meaning that there are potentially multiple locations. Thus, our co-staining data are the most compelling we have seen.

AAK25825/NP_079514, also described as BTB/POZ domain containing 1 (BTBD1), exhibited a cytoplasmic pattern resembling elongated "worm-like" bodies for which we were unable to determine a known structure (data not shown). Members of this domain family have been shown to interact with co-repressor complexes involved in transcriptional repression [20-22]. Previously, BTBD1 has been shown to interact specifically with topoisomerase I [23]. In addition, a recent study further characterized BTBD1 as colocalizing with TRIM family members [24]. Interestingly, TRIM proteins have been shown to exhibit ubiquitin ligase activity.

NP_115683 has no functional annotation and has only been described as an open reading frame located on human chromosome 9. This protein exhibits weak expression when "overexpressed" under control of CMV promoter. Immunofluourescence shows this protein to have both a cytoplasmic and a nuclear distribution. Furthermore, the cytoplasmic expression appears punctate and partially colocalizes with calreticulin, a marker for endoplasmic reticulum (Figure 6C).

AAP34400.1/JC5707 is a member of a family of small C-terminal domain phosphatase (SCP3) and contains an NIF domain (Nuclear Lim Interacting factor-like phosphatase). Other members of this family have been shown to interact with RNA polymerase II and show nuclear localization (SCP1) [25]. Surprisingly, SCP3 was detected at the plasma membrane by colocalization with annexin II, a known component of the plasma membrane (left panel, Figure 3D). In addition to this pattern, we noted fibrous structures that partially colocalized with beta-actin (right panel, Figure 6D).

Hypothetical protein NP_056520 expression was detected by western blot and immunofluorescence microscopy (data not shown). Microscopy detection displayed a cytoplasmic/nuclear rim staining pattern. However, western blot analysis revealed a molecular weight of approximately 50 kDa which is inconsistent with the predicted size of 80 kDa, indicating a possible premature stop in translation or post-translational cleavage. In addition, we were unable to detect ectopic expression for NP_060123. Any attempts to detect this protein via western blot or microscopy were unsuccessful despite sequence validation of the corresponding expression plasmid.

## Conclusion

Specific expression patterns have been identified for the F41D9.1, ZK795.3, C17E4.3 and C09D4.1 GFP-fusion strains. Visible RNAi phenotypes for T24D1.1, F23C8.6, ZK795.3 and B0379.4a were observed. RNAi on T24D1.1a produced an extensive phenotype that was not revealed in the large-scale study by Fraser et al. [5]. Thus, our study shows the value of analyzes focusing on a small number of candidate genes where a variety rather then a pre-defined set of phenotypes are observed. In addition, immunofluorescence microscopy of human cell lines over-expressing NP_219484, NP_848545, AAK25825/NP_079514, NP_115683, AAP34400.1/JC5707 and NP_056520 has detected subcellular localization of the corresponding proteins. The most complete data set comprising both a visible RNAi phenotype or/and a distinct GFP expression in *C. elegans *and a subcellular protein over-expression profile using human cell lines has been obtained for ZK795.3 (NP_219484), F41D9.1 (NP_056520) and C17E4.3 (NP_848515) (Tab. 2).

**Table 2:** The genes for which a visible RNAi phenotype or/and a distinct GFP expression in *C. elegans *and a subcellular protein over-expression profile using human cell lines has been obtained.

**Worm gene / Human ortholog**	**RNAi (C. elegans)**	**GFP expression (C. elegans)**	**Subcellular localization (human)**
T24D1.1 / NP_055733	Smaller population size, sickly appearance, partial sterility, smaller brood size	No specific expression observed	Cloning failed
F23C8.6 / NP_065145	Slow growth, uncoordinated behavior, larval arrest	No specific expression observed	Cloning failed
F38H4.7 / AAK25825	Wild type	No specific expression observed	Cytoplasmic foci
C11D2.4 / NP_115683	Wild type	No specific expression observed	Weak cytoplasmic and nuclear pattern
F41D9.1 / NP_056520	Wild type	neural cells in the head, along the neural nerve cord and in the tail	Cytoplasimic/nuclear rim
ZK795.3 / NP_219484	Slower growth, sterility,	Spermatheca, hypodermal cells, pharynx, excretory cell and channels	Diffuse nuclear, nucleolar, nuclear foci
C17E4.3 / NP_848545	Wild type	Three distinct sheath/socket cells in the head; several cells around the cloaca; pharyngeal muscle cells	Perinuclear/nuclear lamina
B0379.4a / AAP34400	Egg-laying deffects	No specific expression observed	Cytoskeletal/plasma membrane
C09D4.1 /NP_060261	Wild type	Intestinal cells	Protein not detected

In the case of genes F41D9.1 and C17E4.3, a mainly neuronal GFP expression was detected. Therefore, the absence of an observable RNAi phenotype may be explained by the known refractory nature of RNAi in this tissue. For the gene ZK795.3, which showed a more generalized effect on the development, corresponding widespread expression pattern was observed in the worm and in human cell lines. Given that the protein prediction indicates this gene as a snoRNP candidate, its involvement in a variety of cellular processes thus can be expected. To the contrary, restricted subcellular expression patterns in human cells observed in a few cases, for example the AAK25825 (F38H4.7) and NP_115683C11D2.4 genes (Tab. 2), correlate with the absence of phenotypes other than the wild type. This fact suggests that these genes may be of somewhat lesser importance.

In conclusion, this comparative *C. elegans*-human cell lines study based on orthology assignments explores gene functionality by combining two key aspects of functional genomics: a whole organism approach and protein overexpression data using cells lines.

Our results extend currently available information on the selected genes providing a step more towards identifying their exact function.

## Methods

### Phylogenetic analysis

In order to select candidate genes for the RNAi experiments phylogenetic reconstructions were performed as follows: *C. elegans *and related sequences were collected from Genelynx  or/and Wormbase . The data set typically included several homologs of the model organisms (yeast, fly, etc.) and one or more human sequences. Multiple sequences alignments were done using ClustalW [26]. Phylogenetic trees implemented by the SeaView and Phylo_win program [27] were constructed by using maximum parsimony and the neighbor-joining method with 500 bootstrap replicates. Only genes producing well-supported trees (bootstrap value > 50), with the same branch positions, when applying both maximum parsimony and neighbor joining method were selected (Fig. 1.).

### Generation of bacterial feeding library

Total N2 RNA extract was used to synthesize cDNA (Reverse Transcription System; Promega). A pair of oligonucleotides that had restriction sites for *XmaI *(extremely rare sites in the worm genome) was designed for each predicted coding sequence. PCR products of the selected genes were generated using the RT-PCR mixture as template. In addition, primers for spliced leaders SL1 and SL2 were also used. PCR products were ligated into previously digested L4440 (double-T7 vector, Fire lab, ) using Rapid DNA Ligation Kit (Roche). Plasmids were transformed into *E. coli *JM109. Plates were screened for recombinant clones by restriction analysis of plasmid minipreps. Positive clones were grown in overnight cultures. Plasmids were extracted by QIAfilter Plasmid Kit (QIAGEN). Clones were sequenced and used to transform *E. coli *HT115, an RNaseIII-deficient strain used to feed the nematodes.

### RNAi by feeding

RNAi plates were prepared by spreading 200 μl of the bacterial liquid cultures per small (6 cm diameter) NGM plates supplemented with 1 mM IPTG and 25 μg/ml carbenicillin. Plates were seeded with N2 eggs prepared with the standard bleaching method. The hatched worms and their progeny (four generations) were screened for phenotypes other than wild type. As a positive control, the HT115 *E. coli *strain transformed with the *unc-22 *gene ("twitchin") was used (Fire lab vector pPD34.09). N2 strain was incubated at 15 and 25°C. All the experiments were repeated using the NL2099 strain (*rrf-3*) incubated at 15 and 20°C since it is known for a temperature-dependent decrease in the brood size [28,29]. Plates seeded with the empty vector were used as a negative control.

### Gene expression profiles

GFP strains were made by transcriptional fusions of putative promoters (1–2.5 Kb) with GFP using a fusion PCR technique as described by Hubert O. [29].

### Estimation of the life span, brood size, embryonic lethality and duration of the larval stages

NL2099 and N2 worms were grown on plates seeded with bacteria containing double-T7 (L4440) vector without insert and as well as corresponding RNAi plates. Individual F_1 _eggs were transferred onto 10 fresh plates and their development followed at 20°C. Individual worms were transferred every 24 h onto fresh plates. The total number of F_2 _eggs on the plate was counted, as well as the number of eggs that did not hatch. Individual worms were observed daily until they died to ascertain their lifespan.

### Microscopy

Microscopy was carried out with a Zeiss Axioplan system complete with filters for visualizing rhodamine and GFP fluorescence. A Hamamatsu black and white and Zeiss Axiocam colour camera were used for capturing images. Images were processed in Adobe Photoshop. Some images were processed with Openlab 3D restoration software to increase image clarity.

Dye filling experiments were done to assist in identifying cells. Briefly, worms were incubated for an hour in DiI stain and observed with a microscope under UV light through a rhodamine filter. Only amphids and phasmids were stained.

### Exogenous protein expression and immunofluorescence detection in human cell lines

Plasmid constructs containing full-length genes of interest were generated according to previously described methods [30]. Briefly, full-length open reading frames were cloned into a mammalian expression vector containing a CMV promoter and an in-frame N-terminal or C-terminal V5 epitope tag in order to generate fusion proteins for expression studies (pcDNA-DEST40™, Invitrogen). HEK293 and HeLa cells (ATCC) were maintained at 37°C, 5% CO_2 _in Dulbecco's Modified Eagle Medium (DMEM) supplemented with 10% FBS and 100 U/ml Penicillin/Streptomycin (Invitrogen). Cells were seeded on glass coverslips at 80% confluency 24 hours prior to transfection in DMEM containing 10% FBS without antibiotics. Cells were transiently transfected for 24–48 hours with Lipofectamine2000 (Invitrogen) according to the manufacturer's recommendations. Cells were fixed with 2% paraformaldehyde/1.5% sucrose in PBS for 15 minutes at room temperature (RT), after which cells were treated with 0.5% triton-X 100 in PBS for 5 minutes. Cells were incubated for 30 minutes at RT in blocking buffer (2% BSA in PBS) before detection with the primary antibodies. Fusion proteins were detected with either goat anti-V5 (1:500, Bethyl Laboratories) conjugated with a FITC label or an unlabeled mouse anti-V5 (1:200, Invitrogen) for which a secondary detection with goat anti-mouse Alexa 488 (1:1000, Molecular Probes) was performed. Diluted antibodies were applied to coverslips and incubated at 37°C for 2 hours before washing in PBS. Samples were co-stained with the following organelle-specific antibodies and corresponding dilutions: rabbit anti-calreticulin (1:200, Affinity Bioreagents), human anti fibrillarin (1:100, a gift from Dr. N. Ringertz, Karolinska Institute), rabbit anti Lamin (aLi, 1:200, a gift from Dr. G. Simos, EMBL), mouse anti Annexin II (1:250, BD Transduction Laboratories), mouse anti beta-actin (1:500, Sigma). For both the annexin II and the beta-actin antibodies it was necessary to perform Methanol:Acetone (1:1) fixation for 10 minutes instead of paraformaldehyde fixation and triton-x treatment.

Secondary antibody detection was performed at RT for 45 minutes with the following antibodies: anti human-Cy3 (1:1000, Amersham), donkey anti rabbit-Cy3 (1:1000, Jackson), donkey anti mouse-Cy3 (1:1000, Jackson). Coverslips were mounted on slides with Prolong Anti-Fade (Molecular Probes) mounting media. Slides were viewed by Leica DMRA2 and DMRXA microscopes with epifluorescence and images were captured with Openlab™ software version 3.1.4.

## Authors' contributions

IT carried out phylogenetic analysis and experimental work of the *C. elegans *part, and drafted the manuscript. EH carried out protein expression and immunofluorescence detection in human cell lines. PD carried out the microscopy of the *C. elegans *part. RJ contributed in making transgenic strains. AVG conceived of the study and participated in its design and coordination. All authors read and approved the final manuscript.
